# Proportional Borda allocations

**DOI:** 10.1007/s00355-016-0982-z

**Published:** 2016-07-26

**Authors:** Andreas Darmann, Christian Klamler

**Affiliations:** Institute of Public Economics, University of Graz, Graz, Austria

## Abstract

In this paper we study the allocation of indivisible items among a group of agents, a problem which has received increased attention in recent years, especially in areas such as computer science and economics. A major fairness property in the fair division literature is proportionality, which is satisfied whenever each of the *n* agents receives at least $\frac{1}{n}$ of the value attached to the whole set of items. To simplify the determination of values of (sets of) items from ordinal rankings of the items, we use the Borda rule, a concept used extensively and well-known in voting theory. Although, in general, proportionality cannot be guaranteed, we show that, under certain assumptions, proportional allocations of indivisible items are possible and finding such allocations is computationally easy.

## Introduction

In recent years, the problem of fair division has received increased attention, especially in areas such as computer science and economics [see, e.g., the surveys by Bouveret et al. (2016) and Thomson (2016)]. A particularly challenging issue is the fair allocation of indivisible items, which comes up in many real world situations such as divorce settlements, the allocation of courses to students, etc. To evaluate allocation procedures, many intuitive fairness criteria, such as envy-freeness, equitability or proportionality have been used. In the case of divisible items and cardinal utilities, those criteria can be rather easily applied and well known results [e.g., Steinhaus (1948)] show that an envy-free and equitable division of a cake always exists [see also Brams and Taylor (1996)]. In contrast, when we are concerned with indivisible items, even the satisfaction of one of the most basic fairness criterion, proportionality, which requires each of ${n}$ agents to receive at least one ${n}$th of her value of the whole set of items, might be out of reach. The simple example of dividing one single and indivisible item among two agents easily shows the problem.

Different paths of tackling the fair division problem of allocating indivisible items have been taken. Herreiner and Puppe (2002) assume the agents to rank all possible subsets of items and follow a maximin approach, i.e., try to find an allocation of all items to the agents that maximizes the minimum rank of the assigned sets in the preferences of the agents. This approach, however, has two drawbacks. First, the elicitation of such preferences might be an impossible task for the agents, already with a few items (10 items, e.g., lead to the task of ranking more than 1000 sets) and, second, finding the maximin allocation is computationally difficult. To overcome the problem of ranking thousands of sets, many papers only use value information about the single items, mostly incorporating an additivity assumption. This means that the value of any set of items is determined by the sum of the values of the items it contains and therefore no dependencies between items are considered [see, e.g., Brams and Taylor (2000), Bouveret and Lang (2011) and Baumeister et al. (2013)]. Even less information is used when the allocation is based purely on ordinal preference information, e.g., as in Brams et al. (2014) or Aziz et al. (2014). They devise procedures satisfying certain fairness criteria in case a fair allocation exists. In this limited informational framework certain fairness criteria, however, have to be appropriately adapted, sometimes requiring the restriction to situations where the number of items is a multiple of the number of agents or restricting the setting to two agents only.

Probably the most simple approach from an informational perspective is based on picking sequences [see Brams and King (2005) and Bouveret and Lang (2011)] in which the agents pick items according to some pre-defined order.^1^ Finally, Budish (2011) translates the ”I cut you choose” procedure from the cake-cutting literature to the indivisible items setting and focuses on what he calls *maximin shares*, i.e., the minimal share that an agent would receive if she was to divide the items into ${n}$ piles and being the last to pick her pile. This approach was used by Procaccia and Wang (2014) who showed that there is always an allocation that guarantees an agent 2 / 3 of her maximin share.

In the cake-cutting setting, the notion of maximin share is equivalent to the notion of proportionality. This is not the case when we are concerned with the allocation of indivisible items, where proportionality is the stronger property. A general analysis of the differences in various fairness properties for the case of additive preferences has been undertaken by Bouveret and Lemaître (2014), providing a whole scale for fairness properties.

In this paper we focus on the property of proportionality when agents are assumed to have ordinal preferences over a given set of items. The positional information from the ordinal preferences in addition with an additivity assumption will then be used to determine preferences over sets of items. In this setting we show, by using particular picking sequences, that proportionality will—under certain assumptions—be achieved and checking for the existence of such an allocation is computationally easy. To be able to discuss the value of sets of items, we assign Borda scores to the single items determined from positional information in the agents’ preferences. Although its origins stem from voting theory, in which it is widely discussed and analyzed [see Saari (1995)], Borda scores are also used as Borda utilities in welfare economics [see, e.g., Fleurbaey and Hammond (2004)]. The Borda rule—in case of ${k}$ items—assigns ${k}-1$ points to an agent’s top ranked item, ${k}-2$ points to her second ranked item, and so forth, down to zero points for her lowest ranked item. Hence, since we assume additivity, the value of a set of items depends directly on the positions in which the items are ranked by the agent [see, e.g., Brams et al. (2001)].^2^ This seems to be a reasonable approach in case no other than ordinal information is available. Moreover, it could be seen as satisfying an important idea of ordinality, namely that the difference between any two neighboring items in the ranking is identical. Actually, as in addition we assume balanced allocations, i.e., every agent receives the same number of items [and therefore the number of items is a multiple of the number of agents as, e.g., in Brams et al. (2014)], any affine transformation of the Borda scores can be used. As long as the difference between neighboring items remains identical our results do not change. Hence the results are robust for a whole class of scoring rules applied to this setting. Finally, we assume the agents to behave sincerely, i.e., we are not concerned with strategic behaviour of any kind.

The paper is structured as follows: The next section introduces the general framework. Section 3 discusses the general case in which the number of agents is ${n}\ge 2$. This is followed by certain specific results on the two agents case. Section 5 concludes the paper.

## Formal framework

Let $N=\{1,\ldots ,{n}\}$ be a set of ${n}$ agents and let *X* denote a set of ${k}$ items, where ${k}$ is a multiple of ${n}$. Each agent $i\in N$ ranks the items by means of a strict order $\succ _{i}$ over *X*. The agents’ preferences are captured by means of a *preference profile* $(\succ _{1},\succ _{2},\ldots ,\succ _{{n}})$. A mapping $\pi :N\rightarrow 2^{X}$ with $\pi (i)\cap \pi (j)=\emptyset$, $|\pi (i)|=|\pi (j)|$ for each $i,j\in N$ and $\bigcup _{i\in N}\pi (i)=X$ is called *(balanced) allocation*.

A *scoring vector* is a vector ${\omega }=({\omega }^{(1)},{\omega }^{(2)},\ldots ,{\omega }^{({k})})$ of real numbers with ${\omega }^{(j)}\ge {\omega }^{(j+1)}$ for $1\le j\le {k}-1$. The score ${\omega }_{i}(a)$ of item *a* for agent *i* (with respect to ${\omega }$) is given by ${\omega }^{(\rho _{i}(a))}$, where $\rho _{i}(a)$ denotes the rank of item *a* in the ranking $\succ _{i}$. Abusing notation, we define the score of an allocation $\pi$ for agent *i* by ${\omega }_{i}(\pi ):=\sum _{a\in \pi (i)}{\omega }_{i}(a)$.

In particular, *Borda scores* are determined by the scoring vector $({k}-1,{k}-2,\ldots ,1,0)$. The Borda score of item *a* for agent *i* is hence given by $b_{i}(a)=|\{x\in X:x\prec _{i}a\}|$. Again abusing notation, the Borda score of an allocation $\pi$ for agent *i* is given by $b_{i}(\pi ):=\sum _{a\in \pi (i)}b_{i}(a)$.

A picking sequence (or picking protocol) *s* is a sequence of agents serving as a protocol for allocating indivisible items by asking the agents to pick items sequentially following the sequence *s*. Formally, a *picking sequence* *s* is a sequence $s=(s_{1},s_{2},\ldots ,s_{{k}})$ with $s_{i}\in N$ for $1\le i\le {k}$, where at step *i* agent $s_{i}$ picks her most preferred item among those remaining. E.g., with ${k}=6$ and ${n}=3$ sequence $s:=(1,2,3,3,2,1)$ indicates that agent 1 is the first to pick an item, then agent 2 picks an item followed by agent 3 picking two items, next agent 2 picks a second item, and finally agent 1 picks a second item.

Let $V_{{\omega }}({k})=\sum _{j\le {k}}{\omega }^{(j)}$ denote the total score with respect to ${\omega }$ for an agent by getting assigned all of the ${k}$ items. An allocation $\pi$ is *proportional* with respect to ${\omega }$ if ${\omega }_{i}(\pi )\ge \frac{V_{{\omega }}({k})}{{n}}$ holds for each agent $i\in N$.

In what follows, with the exception of Proposition 1, we focus on Borda scores only and proportionality refers to Borda scores exclusively. For the sake of readability, let $V({k})$ denote the total Borda score for an agent by getting assigned all of the ${k}$ items, i.e., $V({k}):=\frac{({k}-1){k}}{2}$. Now, the general question to which the rest of the paper is dedicated is as follows:

Given a set $N=\{1,\ldots ,{n}\}$ of agents, a set *X* of ${k}$ items and a preference profile $(\succ _{1},\succ _{2},\ldots ,\succ _{{n}})$, is there a *proportional* allocation, i.e., an allocation $\pi$ such that $b_{i}(\pi )\ge \frac{V({k})}{{n}}$ holds for each agent $i\in N$?

Note that, by definition, the allocations considered in this work are balanced, i.e., each agent receives the same number of items. As a consequence, proportionality is preserved under any affine transformation of Borda scores.

### Proposition 1

An allocation is proportional for Borda scores if and only if it is proportional for any affine transformation of Borda scores.

### Proof

Let *t* be an affine transformation of Borda scores, i.e., for some $(\alpha ,\beta )\in \mathbb {R}_{\ge 0}\times \mathbb {R}\setminus \{(0,0)\}$ we have $t_{i}(a):=\alpha \cdot b_{i}(a)+\beta$, for each agent $i\in N$ and each $a\in X$. Abusing notation, for allocation $\pi$ and $i\in N$ let $t_{i}(\pi ):=\sum _{a\in \pi (i)}t_{i}(a)$. Let $V_{t}(k)$ denote the total score with respect to *t* for an agent *i* by getting assigned all *k* items, that is

$$\begin{aligned} V_{t}(k)=\sum _{a\in X}t_{i}(a)=\sum _{a\in X}(\alpha b_{i}(a)+\beta )=\left( \alpha \cdot \sum _{a\in X}b_{i}(a)\right) +\beta \cdot k=\alpha \cdot V(k)+\beta \cdot k \end{aligned}$$

Proportionality with respect to *t* would require each agent to receive a set of items with total score of at least $\frac{V_{t}(k)}{n}=\alpha \cdot \frac{V(k)}{n}+\beta \cdot \frac{k}{n}$. Since each agent receives exactly $\frac{k}{n}$ items, for any allocation $\pi$ we have $t_{i}(\pi )=\sum _{a\in \pi (i)}(\alpha b_{i}(a)+\beta )=\alpha \cdot b_{i}(\pi )+\beta \cdot \frac{k}{n}$, for each $i\in N$. Now, clearly $t_{i}(\pi )\ge \frac{V_{t}(k)}{n}\Leftrightarrow b_{i}(\pi )\ge \frac{V(k)}{n}$ holds. Therewith, $\pi$ is proportional w.r.t. Borda scores if and only if it is proportional w.r.t. the affine transformation *t*. $\square$

Proposition 1 allows for two immediate observations. Firstly, it implies that proportionality is preserved under any additive or multiplicative translation of Borda scores. Secondly, w.r.t. proportionality the case of quasi-indifference scores, determined by the scoring vector $(1+\varepsilon (k-1),1+\varepsilon (k-2),\ldots ,1+\varepsilon ,1)$ for some small $\varepsilon >0$, coincides with the case of Borda scores.

### Corollary 1

An allocation is proportional for Borda scores if and only if it is proportional for any additive or multiplicative translation of Borda scores.

### Proof

Additive translations of Borda scores correspond to affine transformations with $\alpha =1$, multiplicative translations of Borda scores correspond to affine transformations with $\beta =0$ (and $\alpha >0$). $\square$

### Corollary 2

An allocation is proportional for Borda scores if and only if it is proportional for quasi-indifference scores.

## Proportional allocations in the general case 

The starting point of our study will be the case in which there are as many items as there are agents [this corresponds to the well known house allocation problem, see for instance Hylland and Zeckhauser (1979)]. The following proposition shows that it is computationally easy to check for the existence of proportionality in that setting.^3^

### Proposition 2

In the case ${k}={n}$, we can check in polynomial time whether a proportional allocation exists and, in case of existence, determine such an allocation.

### Proof

We get $\frac{V({k})}{{n}}=\frac{k(k-1)}{2k}=\frac{k-1}{2}$. Thus, a proportional allocation requires that each agent *i* gets an item *a* with $b_{i}(a)\ge \frac{k-1}{2}$. The problem if such an allocation exists (and if so, to find a proportional allocation) can be solved efficiently by solving the perfect matching problem in the bipartite graph $G=(V,E)$ with $V=N\cup X$ and $E=\{\{i,a\}|i\in N,\,a\in X,\,b_{i}(a)\ge \frac{k-1}{2}\}$. $\square$

Although we can easily check for existence under the restriction ${k}={n}$, it is quite obvious that in many cases such a proportional allocation does not exist at all. The simplest example would be the case of two agents $N=\{1,2\}$ and two items $X=\{a,b\}$. If both agents rank the items in the same way, e.g., $a\succ _{1}b$ and $a\succ _{2}b$, then the allocation of $\pi (1)=\{a\}$ and $\pi (2)=\{b\}$ would lead to $b_{2}(\pi )=0<\frac{V(2)}{2}=\frac{1}{2}$. This violates proportionality. Analogously, assigning the objects in the opposite way would violate proportionality as well. Clearly, this does not only mean that a proportional allocation does not always exist in the case ${k}={n}$; it is also not difficult to see that in general an allocation which is “close” to a proportional allocation (in the sense that the smallest Borda score for an agent is “close” to $\frac{V({k})}{{n}}$) does not exist, because the unanimous profile requires an agent to receive the bottom ranked item yielding a total Borda score of zero.

Things turn out better when we assume ${k}>{n}$, i.e., we have ${k}=\ell \cdot {n}$ for some $\ell >1$, which is done for the rest of this paper. The following result shows that for even $\ell$ we can not only find a picking sequence that provides us with a proportional allocation, but we can also find it quickly.

### Proposition 3

If ${k}=\ell \cdot {n}$ with even $\ell$, then a proportional allocation always exists and can be found in polynomial time.

### Proof

Fix a sequence of length ${n}$ of the ${n}$ agents such that each agent appears exactly once in the sequence, e.g., let the sequence be $(1,2,3,\ldots {n})$. Add the reversal of the sequence to the original sequence, i.e., consider the sequence

$$\begin{aligned} s:=(1,2,3,\ldots ,{n},{n},{n}-1,\ldots ,2,1) \end{aligned}$$

Now, we define a picking sequence *p* by repeating *s* exactly $\frac{\ell }{2}$ times.

We argue that the resulting assignment $\pi$ is proportional. It is sufficient to consider the worst case scenario in which each agent has the same preferences over the items. In such a situation it is easy to verify that each agent receives the same Borda score under $\pi$. Thus, it remains to show that $b_{1}(\pi )\ge \frac{V({k})}{{n}}=\frac{(\ell {n}-1)\ell {n}}{2{n}}=\frac{(\ell {n}-1)\ell }{2}$ holds. We get

$$\begin{aligned} \begin{array}{ccl} b_{1}(\pi ) &{} = &{} [(\ell {n}-1)+(\ell -2){n}]+[(\ell -2){n}-1)+(\ell -4){n}]+\cdots +[(2{n}-1)+0]\\ &{} = &{} \ell {n}-\frac{\ell }{2}+2{n}\sum _{i=1}^{\frac{\ell -2}{2}}2i\\ &{} = &{} \ell {n}-\frac{\ell }{2}+4{n}\frac{\frac{\ell -2}{2}\cdot \frac{\ell }{2}}{2}\\ &{} = &{} \frac{2\ell {n}-\ell +{n}(\ell -2)\ell }{2}\\ &{} = &{} \frac{\ell (\ell {n}-1)}{2} \end{array} \end{aligned}$$

which completes the proof. $\square$

Also in the case of $\ell$ being odd, proportional allocations do exist as long as there is an odd number of agents.

### Theorem 1

Let ${k}=\ell \cdot {n}$ for some $\ell >1$. For an odd number *n* of agents there is always a proportional allocation; such an allocation can be found in polynomial time.

### Proof

By assumption, ${n}$ is odd. If ${k}$ is even, a proportional allocation exists due to Proposition 3. Let ${k}$ be odd, i.e., for some even $\ell$ (or for $\ell =0$), we have ${k}=(\ell +3){n}$. We define a picking sequence *r* by means of two sequences *q*, *s* as follows. For the first $3{n}$ picks, we define the picking sequence *q* by

$$\begin{aligned} q:=(1,\ldots ,{n},{n},{n}-2,{n}-4,\ldots ,1,{n}-1,{n}-3,{n}-5,\ldots ,2,{n}-1,{n}-3,{n}-5,\ldots ,2,{n},{n}-2,{n}-4,\ldots ,1) \end{aligned}$$

The remaining $\ell {n}$ items are picked according to the proof of Proposition 3, i.e., by repeating the picking sequence $s=(1,2,3,\ldots ,{n},{n},{n}-1,\ldots ,2,1)$ exactly $\frac{\ell }{2}$ times. By the proof of Proposition 3 we know that considering only these $\ell {n}$ items, each agent gets a Borda score of at least $\frac{\ell (\ell {n}-1)}{2}$.

Now, we consider the first $3{n}$ items only, allocated according to the picking sequence *q*. Abusing notation, we denote by $b_{i}(q)$ the total Borda score of agent *i* of the items allocated to *i* by applying *q*.

*Let* *i* *be even.* Clearly, the first item allocated to *i* yields a Borda score of at least ${k}-i$, since in the worst case *i* receives her *i*th ranked item. For the second item we need to consider the number of picks before agent *i* picks for the second time, which is $({n}+\frac{{n}+1}{2}+\frac{{n}-i-1}{2})$. Thus, in the worst case as her second item *i* receives her $({n}+\frac{{n}+1}{2}+\frac{{n}-i-1}{2})$th ranked item, yielding a Borda score of ${k}-({n}+\frac{{n}+1}{2}+\frac{{n}-i-1}{2})-1$. Analogously, there are exactly $(2{n}+\frac{{n}-i-1}{2})$ picks before *i* picks her third item. Thus,

1 $$\begin{aligned} \begin{array}{ccl} b_{i}(q) &{} \ge &{} ({k}-i)+{k}- \left( {n}+\frac{{n}+1}{2}+\frac{{n}-i-1}{2}\right) -1+{k}- \left( 2{n}+\frac{{n}-i-1}{2}\right) -1\\ &{} = &{} 3{k}-i-3{n}-({n}-i-1)-\frac{{n}+1}{2}-2\\ &{} = &{} 3{k}-\frac{9{n}+1}{2}-1 \end{array} \end{aligned}$$

In total, we get

2 $$\begin{aligned} {}b_{i}(\pi )\ge & {} 3{k}-\frac{9{n}+1}{2}-1+\frac{\ell (\ell {n}-1)}{2}\nonumber \\= & {} \frac{6(\ell +3){n}-9{n}-2-1+\ell (\ell {n}-1)}{2}\nonumber \\= & {} \frac{6\ell {n}+9{n}-3+\ell ^{2}{n}-\ell }{2}\nonumber \\= & {} \frac{\ell ^{2}{n}+3\ell {n}+3\ell {n}+9{n}-\ell +3}{2}\nonumber \\= & {} \frac{(\ell +3)[(\ell +3){n}-1]}{2}\nonumber \\= & {} \frac{V({k})}{{n}} \end{aligned}$$

*Let* *i* *be odd.* Analogously to above, from the first item *i* receives a Borda score of at least ${k}-i$. The number of picks before agent *i* picks for the second time is $({n}+\frac{{n}-i}{2})$, the number of picks before *i* picks for the third time is $(2{n}+\frac{{n}-1}{2}+\frac{{n}-i}{2})$. Hence,

$$\begin{aligned} \begin{array}{ccl} b_{i}(q) &{} \ge &{} ({k}-i) +\left[ {k}-\left( {n}+\frac{{n}-i}{2}\right) -1\right] +\left[ {k}-\left( 2{n}+\frac{{n}-1}{2}+\frac{{n}-i}{2}\right) -1\right] \\ &{} = &{} 3{k}-i-3{n}-({n}-i)-1-\frac{{n}-1}{2}-1\\ &{} = &{} 3{k}-\frac{9{n}+1}{2}-1 \end{array} \end{aligned}$$

Analogously to above, $b_{i}(\pi )\ge \frac{V({k})}{{n}}$ follows. $\square$

On the negative side, for odd $\ell$ a proportional allocation does not always exist if the number of agents is even. In particular, this is the case when the agents have unanimous preferences.

### Theorem 2

Let ${k}=\ell \cdot {n}$ for some odd $\ell$. If the number ${n}$ of agents is even and all agents have identical preferences, then a proportional allocation does not exist.

### Proof

Proportionality requires each agent to receive a set of items with total Borda score of at least

3 $$\begin{aligned} \frac{V(k)}{n}=\frac{1}{n}\frac{(\ell n-1)\ell n}{2}=\frac{(\ell n-1)\ell }{2} \end{aligned}$$

Due to $\ell$ being odd and *n* being even, $(\ell n-1)$ is an odd number. Consequently, $(\ell n-1)\ell$ is odd. Since the Borda score is obviously integer-valued, proportionality requires that each agent receives a set of items with total Borda score of at least $\frac{(\ell n-1)\ell }{2}+\frac{1}{2}$. Clearly, identical preferences thus imply that $V(k)\ge n(\frac{(\ell n-1)\ell }{2}+\frac{1}{2})$ holds which contradicts with (3).$\square$

Although we cannot guarantee proportionality in all cases, we can show that there is a limit to how “far away” we are from such a proportional allocation. In particular, the guaranteed value of the set of items to any agent will not be lower than the largest integer smaller than $\frac{V({k})}{{n}}$ even in the situation in which all agents have identical preferences.

### Theorem 3

Let ${k}=\ell \cdot {n}$ for some $\ell >1$. There is an allocation $\pi$ with $b_{i}(\pi )\ge \left\lfloor \frac{V({k})}{{n}}\right\rfloor$ for each agent *i* that can be found in polynomial time.

### Proof

By Theorem 1, for an odd number of agents a proportional allocation always exists and can be determined quickly. Therefore, we only need to consider the case of an even number of agents.

By Proposition 3, if $\frac{{k}}{{n}}$ is even, then a proportional allocation exists. It remains to consider the case that $\frac{{k}}{{n}}$ is odd. Recall that by assumption $\frac{{k}}{{n}}>1$ holds. I.e., ${k}=(\ell +3){n}$ for some even $\ell$ or for $\ell =0$. Apply the following picking sequence $r'$, defined in similar fashion as the sequence *r* in the proof of Theorem 1.

For the first $3{n}$ picks, let $r'$ be defined by

$$\begin{aligned} q':=(1,2,\ldots ,{n},{n},{n}-2,{n}-4,\ldots ,2,{n}-1,{n}-3,{n}-5,\ldots ,1,{n}-1,{n}-3,{n}-5,\ldots ,1,{n},{n}-2,{n}-4,\ldots ,2) \end{aligned}$$

For the remaining picks, repeating the picking sequence $s=(1,2,3,\ldots ,{n},{n},{n}-1,\ldots ,2,1)$ exactly $\frac{\ell }{2}$ times.

Note that ${n}$ is even, i.e., $\frac{V({k})}{{n}}=\frac{(\ell +3)[(\ell +3){n}-1]}{2}$ is not an integer. In particular,

4 $$\begin{aligned} \left\lfloor \frac{V({k})}{{n}}\right\rfloor =\frac{V({k})}{{n}}-\frac{1}{2} \end{aligned}$$

holds.

*Let* *i* *be even.* Clearly, the first item allocated to *i* yields a Borda score of at least ${k}-i$. For the second item, the number of picks before agent *i* picks for the second time is $({n}+\frac{{n}-i}{2})$. In the worst case, *i* hence receives her $({n}+\frac{{n}+1}{2}+\frac{{n}-i-1}{2})$th ranked item with a Borda score of ${k}-({n}+\frac{{n}-i}{2})-1$. Next, there are exactly $(2{n}+\frac{{n}}{2}+\frac{{n}-i}{2})$ picks before *i* picks her third item. Thus,

$$\begin{aligned} \begin{array}{ccl} b_{i}(q) &{} \ge &{} ({k}-i)+{k}- \left( {n}+\frac{{n}-i}{2}\right) -1+{k}-\left( 2{n}+\frac{{n}}{2}+\frac{{n}-i}{2}\right) -1\\ &{} = &{} 3{k}-i-3{n}-({n}-i)-\frac{{n}}{2}-2\\ &{} = &{} 3{k}-\frac{9{n}}{2}-2 \end{array} \end{aligned}$$

Similarly to (2), we get

$$\begin{aligned} \begin{array}{ccl} b_{i}(\pi ) &{} \ge &{} 3{k}-\frac{9{n}}{2}-2+\frac{\ell (\ell {n}-1)}{2}\\ &{} = &{} \frac{6(\ell +3){n}-9{n}-4+\ell (\ell {n}-1)}{2}\\ &{} = &{} \frac{6\ell {n}+9{n}-3+\ell ^{2}{n}-\ell }{2}-\frac{1}{2}\\ &{} = &{} \frac{(\ell +3)[(\ell +3){n}-1]}{2}-\frac{1}{2}\\ &{} = &{} \frac{V({k})}{{n}}-\frac{1}{2}\\ &{} = &{} \left\lfloor \frac{V({k})}{{n}}\right\rfloor \end{array} \end{aligned}$$

where the last equality follows from (4).

*Let* *i* *be odd.* The first item allocated to *i* has a Borda score of at least ${k}-i$. For the second item, the number of picks before *i* picks her second item is $({n}+\frac{{n}}{2}+\frac{{n}-i-1}{2})$. Finally, there are exactly $(2{n}+\frac{{n}-i-1}{2})$ picks before she can pick her third item. Thus,

$$\begin{aligned} \begin{array}{ccl} b_{i}(q) &{} \ge &{} ({k}-i)+{k}-\left( {n}+\frac{{n}}{2}+\frac{{n}-i-1}{2}\right) -1+{k}-\left( 2{n}+\frac{{n}-i-1}{2}\right) -1\\ &{} = &{} 3{k}-i-3{n}-({n}-i-1)-\frac{{n}}{2}-2\\ &{} = &{} 3{k}-\frac{9{n}}{2}-1 \end{array} \end{aligned}$$

Hence, we get

$$\begin{aligned} \begin{array}{ccl} b_{i}(\pi ) &{} \ge &{} 3{k}-\frac{9{n}}{2}-1+\frac{\ell (\ell {n}-1)}{2}\\ &{} = &{} \frac{6(\ell +3){n}-9{n}-2+\ell (\ell {n}-1)}{2}\\ &{} = &{} \frac{6\ell {n}+9{n}-3+\ell ^{2}{n}-\ell }{2}+\frac{1}{2}\\ &{} = &{} \frac{(\ell +3)[(\ell +3){n}-1]}{2}+\frac{1}{2}\\ &{} = &{} \frac{V({k})}{{n}}+\frac{1}{2}\\ &{} = &{} \left\lceil \frac{V({k})}{{n}}\right\rceil \end{array} \end{aligned}$$

which completes the proof. $\square$

The previous results show that there are—so far—only a couple of cases in which proportionality cannot be guaranteed, namely those with ${n}$ being even and $\ell$ being odd. For the case of ${n}$ being even we now show that, whenever ${n}\ge 4$, proportionality can still be achieved as long as preferences differ from the unanimous preference profile by a certain distance. For that purpose, distance is measured by swaps of items.

In that respect, given a ranking $\succ$ over a set $X=\{x_{1},x_{2},\ldots ,x_{{k}}\}$, let $o_{\succ }(\ell )$ denote the $\ell$-th ranked element in the ranking $\succ$, $1\le \ell \le {k}$. For two rankings $\succ ,\succ '$ over a set $X=\{x_{1},x_{2},\ldots ,x_{{k}}\}$ we say $\succ '$ *corresponds to* $\succ$ *up to a swap in rank* *j* if we have $o_{\succ }(j)=o_{\succ '}(j+1)$, $o_{\succ }(j+1)=o_{\succ '}(j)$, and for all $\ell \in \{1,2,\ldots ,{k}\}\setminus \{j,j+1\}$ it holds that $o_{\succ }(\ell )=o_{\succ '}(\ell )$. The following theorem states that a proportional allocation exists even in the case that half of the agents have the same ranking $\succ$, and, for some *h*, the rankings of the remaining agents are given by those that correspond to $\succ$ up to a swap in position $h+g$, $g\in \{0,1,\ldots ,\frac{n}{2}-1\}$.

### Theorem 4

Let ${k}=\ell \cdot {n}$ for some $\ell >1$. Let ${n}\ge 4$ be even, $S\subset N$ with $|S|=\frac{{n}}{2}$ and let $\succ$ be a ranking over *X*.

If the preference profile $(\succ _{1},\succ _{2},\ldots ,\succ _{{n}})$ is such that for all $i\in S$ we have $\succ _{i}=\succ$, and for some $h\ge 1$ there are a set $C_{h}=\{h,h+1,\ldots ,h+\frac{{n}}{2}-1\}$ and a bijection $f:N\setminus S\rightarrow C_{h}$ such that $\succ _{i}$ corresponds to $\succ$ up to a swap in rank *f*(*i*), then there is a proportional allocation.

### Proof

Again, we can assume that ${k}=\ell {n}$ for some odd $\ell >1$. We assume ${k}=3{n}$, the remaining cases follow analogously. In what follows, we will consider the sequence $q'$ defined in the proof of Theorem 3. We call an agent odd/even if the agents’ label is odd/even.

The goal is to modify $q'$ such that for the profile considered each odd agent gets the same total Borda score as in $q'$ for the unanimous profile, while each even agent’s Borda score increases by 1. Let *j* be the agent whose ranking $\succ _{j}$ corresponds to $\succ$ up to a swap in rank *h*.

First, we assume $h>{n}$ (the case $h\le {n}$ will follow analogously), and label $\frac{{n}}{2}$ agents according to $C_{h}$: for $i\in C_{h}$, $q'_{i+1}$ is the agent with the swap in rank *i* ($q'_{i}$ denotes the $i'$th agent in the sequence $q'$). The remaining agents (each of whose ranking coincides with $\succ$) are labeled in arbitrary manner such that finally the set of agents is given by $\{1,\ldots ,{n}\}$. Relabel the items such that for the ranking $\succ$ we have $x_{1}\succ x_{2}\succ \cdots \succ x_{3{n}}$.

- Case 1: $h\in \{{n}+1,\ldots ,\frac{3{n}}{2}-1\}$. I.e., $q'_{h+1}=i$ for some even $i\ge 2$. Consider the picking sequence $q'$. Note that, for the item picked in pick round $h+1$, agent *i* receives a Borda score of $3{n}-h$ (instead of $3{n}-h-1$ in the case of unanimous profile), because—due to the swap—she receives the item $x_{h+1}$ of rank *h* in the ranking $\succ _{i}$. Note that so far all the items $\{x_{1},x_{2},\ldots ,x_{h+1}\}$ have been picked. Analogously, in the next step, the next picking agent *j* receives item $x_{h+2}$ of rank $h+1$ in $\succ _{j}$, etc. I.e., each agent picking in rounds $h+1,h+2,\ldots ,h+\frac{{n}}{2}+1$ ends up with an additional score of 1 when compared to $q'$ and the unanimous profile case. By assumption, *i* is even. Considering $q'$ we can conclude that the odd agents picking in the above-mentioned rounds are the agents ${n}-1,{n}-3,\ldots ,i+1$. Now, modify the sequence $q'$ in the first ${n}$ picks by “swapping” the pick round of each of these odd agents with its even successor, leaving the remaining picking sequence unchanged:
  $$\begin{aligned} \tilde{q}=(1,2,\ldots ,i-1,i,i+2,i+1,i+4,i+3,\ldots ,{n},{n}-1) \end{aligned}$$
  Now, define the picking sequence $q''$ by picking according to $\tilde{q}$ for the first ${n}$ rounds and then continuing with picking according to $q'$, i.e.,
  $$\begin{aligned} q''_{h}={\left\{ \begin{array}{ll} \tilde{q}_{h} \quad h\le {n}\\ q'_{h} \quad h>{n}\end{array}\right. } \end{aligned}$$
  It is not hard to see that compared with the Borda scores under $q'$ for the unanimous profile, in the considered profile $q''$ increases the Borda score of the even agents by 1 while the Borda score of the odd agents remains unchanged. I.e., the resulting allocation is proportional.
- Case 2: $h\in \{\frac{3{n}}{2},\frac{3{n}}{2}+1,\cdots ,\frac{5{n}}{2}-1\}$. Again, let us consider the picking sequence $q'$. Analogously to above, all agents $q'_{h+1},q'_{h+2},\ldots ,q'_{h+\frac{{n}}{2}}$ end up with an increase of Borda score of 1. If all these agents are even (i.e., $h=\frac{5{n}}{2}-1$), $q'$ yields a proportional allocation and we are done.
  - Case 2a. $q'_{h+1},q'_{h+2},\ldots ,q'_{h+\frac{{n}}{2}}$ are all odd. Modify the picking sequence $q'$ in the first ${n}$ rounds by “swapping” the pick round of all odd agents with its even successor:
    $$\begin{aligned} \hat{q}=(2,1,4,3,\ldots ,{n},{n}-1) \end{aligned}$$
    Pick according to $\hat{q}$ for the first ${n}$ rounds and then continue according to $q'$, i.e.,
    $$\begin{aligned} q'''_{h}={\left\{ \begin{array}{ll} \hat{q}_{h} &{} h\le {n}\\ q'_{h} &{} h>{n}\end{array}\right. } \end{aligned}$$
    When compared with the Borda scores under $q'$ for the unanimous profile, it again follows that $q'''$ increases the Borda score of the even agents by 1 while the Borda score of the odd agents remains unchanged. Thus, the resulting allocation is proportional.
  - Case 2b. Some of $q'_{h+1},q'_{h+2},\ldots ,q'_{h+\frac{{n}}{2}}$ are even. These even agents must be the agents ${n},{n}-2,\ldots ,j+3$. For the remaining even agents, modify the picking sequence $q'$ in the first ${n}$ rounds by “swapping” the pick round of each of these agents with its odd predecessor:
    $$\begin{aligned} \bar{q}=(2,1,4,3,\ldots ,j+1,j,j+2,j+3,\ldots ,{n}-1,{n}) \end{aligned}$$
    Pick according to $\bar{q}$ for the first ${n}$ rounds and then continue picking according to $q'$, i.e.,
    $$\begin{aligned} q_{h}''''={\left\{ \begin{array}{ll} \bar{q}_{h} \quad h\le {n}\\ q'_{h} \quad h>{n}\end{array}\right. } \end{aligned}$$
    Again, it is not hard to see that that the Borda score of each agent has increased by 1 while the Borda score of the odd agents has remained unchanged when compared to $q'$ applied on the unanimous profile. Hence, the resulting allocation is proportional.

If $h\le {n}$, then instead of $q'$ consider the picking sequence $p'$, which can be seen as a modification of $q'$ which simply delays the first ${n}$ picks of $q'$ to the last ${n}$ picks:

$$\begin{aligned} p':= & {} ({n},{n}-2,\ldots ,2,{n}-1,{n}-3,\ldots ,1,\\&{n}-1,{n}-3,\ldots ,1,{n},{n}-2,\ldots ,2,1,2,\ldots ,{n}) \end{aligned}$$

It is not hard to verify that also $p'$ satisfies $b_{i}(p')\ge \left\lfloor \frac{V({k})}{{n}}\right\rfloor$ for each agent *i*. Now, in the case of $h\le {n}$ arguing analogously as in the case $h>{n}$ with $p'$ instead of $q'$ (and the modification taking place in the last ${n}$ pick rounds) yields the desired result.

Finally, it is not hard to see that the case ${k}=\ell {n}$ for odd $\ell \ge 5$ and even ${n}\ge 4$ follows analogously. The idea is to allocate $3{n}$ items (determined by the rank *h* where the first swap occurs) according to above and the remaining items by repeated application of the picking sequence $s=(1,2,3,\ldots ,{n},{n},{n}-1,\ldots ,2,1)$. In particular, let *m* be the smallest non-negative integer such that $2{n}m+3{n}\ge (h+\frac{{n}}{2}-1)$ holds. In the first $2{n}m$ pick rounds let the agents pick by repeating *s* exactly *m* times. The next $3{n}$ items are picked just as in the case ${k}=3{n}$ described above. Finally, the remaining items are picked by repeatedly applying *s* as long as there are items left. $\square$

## Proportional allocations in the two agents case

Many fair division problems focus on the more specific case of two agents, i.e., ${n}=2$ [see, e.g., Brams and Taylor (2000) and Brams et al. (2012, (2014)]. Although we were able to show that a proportional allocation always exists whenever every agent is assigned an even number of items, at the beginning of the previous section we provided a simple example for $\ell =1$ where proportionality was violated. This can be extended to any odd $\ell$ whenever the agents have identical preferences. Consider the case of $\ell =3$, with $X=\{a,b,c,d,e,f\}$ and, w.l.o.g., preferences of the following form, where items are ranked from top to bottom:

$$\begin{aligned} \begin{array}{c|c} \succ _{1} &{} \succ _{2}\\ \hline a &{} a\\ b &{} b\\ c &{} c\\ d &{} d\\ e &{} e\\ f &{} f \end{array} \end{aligned}$$

Obviously, $V(6)=15$, and any division of the total value of 15 will assign a set of items to one agent being valued below the proportionality threshold of $\frac{V(6)}{2}=7.5$.^4^ As for $\ell$ being odd and identical preferences, the total maximal Borda score will always be an odd number, it is clear that no proportional allocation is possible in such cases. However, even the slightest difference in the preferences of the agents is sufficient to guarantee proportionality again.

### Theorem 5

Let $\ell \not =3$. For $2\ell$ items and two agents 1, 2 with $\succ _{1}\not =\succ _{2}$, a proportional allocation always exists and can be found quickly.

### Proof

The case $\ell =1$ is obvious, the case $\ell$ even follows from Proposition 3. Let $\ell$ be odd, $\ell >3$. We consider the case $\ell =5$ first. We need to show that each agent’s Borda score is at least $\left\lceil \frac{9\cdot 10}{2\cdot 2}\right\rceil =23$.

Let *h* denote the minimal rank in which the *h*th ranked items in $\succ _{1}$ and $\succ {}_{2}$ do not coincide (clearly, $h\le 9$ holds).

- Case 1: $h\in \{2,5,7,9\}$. Consider the picking sequence $r_{1}=(1,2,1,2,2,1,2,1,2,1)$. Clearly, in each picking round *g*, the picking agent in the worst case picks her *g*th ranked item. Thus, $b_{2}(r_{1})\ge 8+6+5+3+1=23$. In addition, $b_{1}(r_{1})\ge (9+7+4+2+0)+1=23$, because in round $h+1$, agent 1 can pick her *h*th ranked item instead of her $(h+1)$th ranked item, gaining an additional Borda score of 1.
- Case 2: $h\in \{1,8\}$. Consider the picking sequence $r_{2}=(1,2,2,2,1,1,1,1,2,2)$. Then $b_{1}(r_{2})\ge 9+5+4+3+2=23$ and $b_{2}(r_{2})\ge (8+7+6+1+0)+1=23$, since in round $h+1$, agent 2 now picks her *h*th ranked item instead of her $(h+1)$th ranked item, gaining an extra Borda score of 1.
- Case 3: $h=6$. With the picking sequence $r_{3}=(1,2,1,2,2,2,1,1,1,2)$ we get $b_{2}(r_{3})\ge 8+6+5+4+0=23$ and $b_{1}(r_{3})\ge 9+7+4+2+1=23$, because in round 7 agent 1 can pick her sixth ranked item with Borda score 4.
- Case 4: $h=3$. The sequence $r_{4}=(1,2,2,1,1,2,2,1,2,1)$ yields $b_{2}(r_{4})\ge 8+7+4+3+1=23$ and $b_{1}(r_{4})\ge 9+7+5+2+0=23$, since in round 4 agent 1 can pick her third ranked item with Borda score 7.
- Case 5: $h=4$. The sequence $r_{5}=(1,2,2,1,2,1,1,2,1,2)$ yields $b_{1}(r_{5})\ge 9+6+4+3+1=23$ and $b_{2}(r_{5})\}\ge 8+7+6+2+0=23$ because in round 5 agent 2 picks her fourth ranked item with Borda score 6.

Consider odd $\ell >5$. Let $h^{*}=2\ell -9$. For $h\ge h^{*}$, we can argue analogously to above, letting the sequence for the last 10 picks be determined according to the five cases above (with $h=h^{*}+i$ instead of $h=i$, $i\in \{1,\ldots ,9\}$, determining the respective case), while the first $2\ell -10$ rounds are picked according to $(1,2,2,1,1,2,2,1,\ldots ,1,2,2,1)$, i.e., repeating (1, 2, 2, 1) exactly $\frac{2\ell -10}{4}$ times. It is not hard to see that this results in a proportional allocation.

For $$h\le h^{*}$$, note that $$2\ell =2+4j$$ for some $$j\in \mathbb {N}$$. Consider the sequence $$p^{*}=(1,2,2,1,1,2,2,1,\ldots ,1,2,2,1,1,2)$$. It is not hard to verify that $$b_{1}(p^{*})\ge \left\lceil \frac{V(2\ell )}{2}\right\rceil $$ and $$b_{2}(p^{*})\ge \left\lfloor \frac{V(2\ell )}{2}\right\rfloor $$ holds (see Theorem 3). Now, if *h* is such that in picking round *h* it is agent 1’s turn while in round $$h+1$$ it is agent 2’s turn, then obviously $$b_{2}(p^{*})\ge \left\lfloor \frac{V(2\ell )}{2}\right\rfloor +1=\left\lceil \frac{V(2\ell )}{2}\right\rceil $$ holds; i.e, $$p^{*}$$ yields a proportional allocation. If in round *h* it is agent 2’s turn and in $$h+1$$ it is agent 1’s turn, then consider the sequence $$p^{**}=(1,2,2,1,1,2,2,1,\ldots ,1,2,2,1,2,1)$$. Analogously to above it follows that $$p^{**}$$ yields a proportional allocation.

If in both *h* and $h+1$ it is agent 1’s turn, modify $p^{*}$ into $\tilde{p}$ by letting agent 2 pick in rounds $h+1$ and $2\ell -2$, and 1 in rounds $h+2$ and $2\ell -3$. $\tilde{p}$ yields a proportional allocation because $b_{1}(\tilde{p})\ge \left\lceil \frac{V(2\ell )}{2}\right\rceil -1+1$ and $b_{2}(\tilde{p})\ge \left\lfloor \frac{V(2\ell )}{2}\right\rfloor +2-1=\left\lceil \frac{V(2\ell )}{2}\right\rceil$.

Finally, if in both rounds *h* and $$h+1$$ it is agent 2’s token, modify $$p^{**}$$ into $$\hat{p}$$ by letting agent 1 pick in rounds $$h+1$$ and $$2\ell -4$$, and agent 2 in rounds $$h+2$$ and $$2\ell -5$$. With $$b_{2}(\hat{p})\ge \left\lceil \frac{V(2\ell )}{2}\right\rceil -1+1$$ and $$b_{1}(\hat{p})\ge \left\lfloor \frac{V(2\ell )}{2}\right\rfloor +2-1=\left\lceil \frac{V(2\ell )}{2}\right\rceil $$ it follows that $$\tilde{p}$$ yields a proportional allocation.$$\square $$

Finally, this leaves us with the case $\ell =3$ where not only the identical preferences case is problematic but there are a couple of more profiles in which proportionality cannot be guaranteed. In particular, there are four such cases as shown in the following result:

### Theorem 6

For two agents and six items there are exactly four cases in which a proportional allocation does not exist.

### Proof

It is a tedious but not difficult task to show that in the following four cases (we fix the ranking of agent 1 to $a\succ b\succ c\succ d\succ e\succ f$ (in short: *abcdef*) and give the ranking of agent 2 only) a proportional allocation does not exist: *abcdef*, *abcedf*, *acbdef*, *acbedf*

For the remaining cases, we need to show the existence of a proportional allocation, i.e., an allocation yielding a score of at least $\frac{V(6)}{2}=\frac{30}{4}=7.5$, i.e., of 8 for each agent. Again, let *h* denote the minimal rank in which the *h*th ranked items in $\succ _{1}$ and $\succ {}_{2}$ do not coincide. If $h\in \{1,5\}$, then $p=(1,2,2,1,1,2)$ yields $b_{1}(p)\ge 5+2+1=8$ and $b_{2}(p)\ge (4+3+0)+1=8$, where the additional score of 1 of agent 2 comes from picking the item ranked *h* in round $h+1$. If $h=3$, then the sequence $p'=(1,2,2,1,2,1)$ yields $b_{1}(p)\ge 5+3+0=8$ and $b_{2}(p)\ge 4+3+1=8$.

Let $h=2$. Let *y* denote the item ranked second by agent 2. Clearly, $y\notin \{a,b\}$ holds due to $h=2$.

*Case 1:* $y\in \{d,e,f\}$. Choose $x\in \{d,e\}\setminus \{y\}$ and let $z\in \{d,e,f\}\setminus \{x,y\}$. $\pi$ assigns the items *a*, *y*, *z* to agent 2, and the remaining items *b*, *c*, *x* to agent 1. Then, $b_{1}(\pi )\ge 4+3+1=8$ and $b_{2}(\pi )\ge 5+4+0=9$.

*Case 2:* $$y=c$$.

*Case 2a:* The third-ranked item of agent 2 is not item *b*. Consider the picking sequence $p=(1,1,2,2,2,1)$. Note that in round 3 agent 2 can pick her second-ranked item (due to $h=2$), and in round 4 agent 2 can pick her third-ranked item (since this item does not coincide with item *a* or *b*). Thus, $b_{2}(p)\ge 4+3+1=8$ while $b_{1}(p)\ge 5+4=9$ holds.

*Case 2b:* The third-ranked item of agent 2 is item *b*. For the rankings *acbdef*, *acbedf* we already know that a proportional allocation does not exist. Hence, it remains to consider the cases *acbdfe*, *acbefd*, *acbfde* and *acbfed*. For each of these cases $\pi$ assigns *b*, *c*, *f* to agent 2 and *a*, *d*, *e* to agent 1. It is easy to verify that $\pi$ is proportional in each of these cases.

Let $h=4$. For agent 2’s ranking being *abcedf* we already know that a proportional allocation does not exist. Therewith, it remains to consider the cases that agent 2’s ranking corresponds to one of the following: *abcefd*, *abcfde*, *abcfed*. Again, the assignment $\pi$ which assigns *b*, *c*, *f* to agent 2 and *a*, *d*, *e* to agent 1 is proportional in each of these cases. $\square$

## Conclusion

This paper has discussed the fairness property of proportionality in the allocation of indivisible items. To use the concept of proportionality we assumed the Borda rule as a scoring method to determine values of items and sets. In general, the decision problem whether there is an allocation such that the Borda score of each agent exceeds a given lower bound is known to be computationally difficult (Baumeister et al. 2013). For the case of identical preferences and the use of Borda scores, (Bouveret and Lang 2011) propose dynamic programming to solve that problem (which hence can be applied for checking for proportionality), which allows a polynomial running time only under unary encoding. However, using additional restrictions on the general setting, such as the number of items being a multiple of the number of agents, in almost all preference profiles not only the existence of a proportional allocation can be assured, but also the determination of such an allocation becomes computationally easy. In addition, to show this, rather simple picking sequences are used. Our proofs are mostly starting with identical preferences determining the worst case, hence the realistic case in which there is at least some difference in the agents’ preferences makes it almost certain to guarantee proportionality based on Borda score valuation.

One could of course argue that the assumption of balanced allocations is too restrictive. Actually, in certain cases, even if we have identical preferences, an unbalanced allocation can lead to proportional shares. As an example, consider three agents having the same ranking $a\succ b\succ c\succ d\succ e\succ f$ over six items. Then the unbalanced allocation $\pi (1)=\{a\}$, $\pi (2)=\{b,e\}$ and $\pi (3)=\{c,d,f\}$ is proportional as is the balanced allocation $\pi '(1)=\{a,f\}$, $\pi '(2)=\{b,e\}$ and $\pi '(3)=\{c,d\}$. However, there are two arguments in favor of the restriction to balanced allocations. First, while proportionality, if satisfied for the usual Borda scores, is preserved under any affine transformation of that scores in the case of balanced allocations, this is in general not the case for unbalanced allocations. For example, in the above situation, any additive transformation of the Borda scores (which in voting theory does not change the outcome of the Borda rule) leads to a violation of proportionality by the unbalanced allocation. Second, most of our results can be seen as possibility results, i.e., they would also be true in case we allow for a broader class of allocations as long as balanced allocations would still be possible. Of course the opposite is true for results in which balanced allocations do not lead to the desired outcome such as in the case of an even number *n* of agents and odd $\ell$, with a total number of $\ell \cdot n$ items. In that case, if the agents have identical preferences, a proportional allocation does not exist, even if we allow for unbalanced allocations (see Theorem 2 and its proof). For $n=2$ and $\ell$ odd, however, a proportional allocation exists if and only if a proportional balanced allocation exists; hence, there are even no unbalanced allocations which are proportional whenever there are no balanced allocations which are proportional. For even $n>2$, $\ell$ odd and non-unanimous preferences this is still an open question.

Of course there is sufficient scope for additional work. First, it would be interesting to know how to handle situations in which the number of items is not a multiple of the number of agents. It is unclear whether picking sequences could still be used in that case. Most likely certain domain restrictions could still guarantee proportionality, but those are yet to be found. Second, the results cannot easily be extended to the class of all scoring functions. The Borda rule has been shown to be special among this class when used in voting theory [see Saari (1995)], but it also seems particularly useful when considered in the allocation of indivisible items. Again, domain restrictions might do the trick to also find positive results for other scoring functions. Third, other fairness properties could be considered. Using Borda scores as numerical values for sets of items, envy-freeness could also be analyzed. However, as envy-freeness is a stronger property for ${n}\ge 3$, no such clear-cut results are to be expected. Finally, as we were only concerned with sincere behaviour, the influence of strategic behaviour on achieving proportionality might be of interest.

## Footnotes

1
Budish and Cantillon (2012) call it the *draft mechanism* and analyze its strategic aspects [see also Kalinowski et al. (2013)].

2
Caragiannis and Procaccia (2011) analyze the distortions induced by this scoring approach stating that these are—in general—rather low.

3 Note that Proposition 2 in fact is not restricted to Borda scores but holds for all scoring rules (that allow for a polynomial-time computation of the score of an item for an agent).

4 Beware though, that we can still find an allocation $\pi$ for which $b_{i}(\pi )\ge 7$ for $i\in \{1,2\}$ as shown in Theorem 3.
